# mTORC1 regulates nutrient access in Ras-mediated tumors

**DOI:** 10.18632/aging.100974

**Published:** 2016-06-09

**Authors:** Heesun Cheong

**Affiliations:** Biomedicine Research Branch, Division of Cancer Biology, National Cancer Center, Goyang, Gyeonggi, 410-769, Republic of Korea

**Keywords:** mTORC1, autophagy, macropinocytosis, Ras

Cancer cells generally undergo metabolic re-programming to satisfy greater metabolic demands for rapid proliferation, thereby shifting toward anabolic conditions. In addition, cancer cells engage in alternative metabolic pathways to deal with various metabolic stresses, which include unfavorable nutrient and oxygen status due to rapid cell growth and accumulation of unnecessary metabolic byproducts derived from metabolic rewiring [1].

Accumulating evidence suggests that cancer cells also utilize other pathways for macromolecule degradation to efficiently obtain necessary nutrients and maintain intact metabolism. In particular, autophagy, a typically lysosome-mediated catabolic process, plays a tumor-promoting role in established cancer by providing intracellular metabolites to cells through the degradation of cellular components to support cell growth and survival [2, 3].

Although oncogenic signals suppress catabolic pathways such as autophagy, and facilitate strong anabolic conditions, autophagic activity is significantly enhanced in oncogenic Ras driven-cancers. Moreover, oncogenic Ras-expressing cells show elevated levels of macropinocytosis, a type of endocytosis in which cells take up extracellular fluid and fluidal components. Consequently, internalized extracellular macro-molecules are degraded in a lysosome-mediated manner, which might confer the availability of nutrients to support cell growth under conditions of metabolic stress [4].

In particular, pancreatic ductal adenocarcinoma (PDAC) cells harboring oncogenic Ras mutant as a major oncogenic driver were found to increase macropinocytic degradation of bovine serum albumin (BSA) from extracellular fluid and supply nutrients, such as amino acids, for sustaining cell growth and survival under dedicated nutrient-deprived conditions. As evidence of this unique metabolic process, active macropinocytosis driven by oncogenic Ras is observed in primary human PDAC tissue, which also shows distinct types of amino acids generated from extracellular proteins as compared with adjacent normal tissue [5].

Recently, we and another group reported that mTORC1, which is affected by environmental nutrient status, is a regulatory mechanism of macropinocytosis [6, 7]. The inactive status of mTORC1 under nutrient-starved conditions can be restored by treatment with albumin from the extracellular environment, whereas albumin-induced mTORC1 reactivation is abolished by inhibiting macropinocytosis and lysosomal degradation. The catabolic degradation of albumin might produce critical nutrients including essential amino acids and glutamine, which enable mTORC1 activation and the proliferation of cells under nutrient-starved conditions.

mTORC1 activity can be assessed by the phosphory-lation levels of mTORC1 substrates and the subcellular localization of mTORC1. The phosphorylation levels of p70 S6K1 and ribosomal protein S6, which decrease in amino acid-deficient conditions, rebound after the addition of extracellular albumin. Inactive mTORC1 is localized in the cytoplasm under amino acid-deprived conditions, but the presence of albumin leads to the localization of mTORC1 at the lysosomal membrane.

This reactivation of mTORC1 by albumin supplementation is impaired by inhibited autophagy. Albumin restores the phosphorylation of mTORC1 substrates in wild-type cells but not in cells deficient in the autophagy-related gene Atg5. Accordingly, not only lysosomal function, but also autophagy plays a critical role in degrading macropinocytic cargo molecules such as albumin. Moreover, oncogenic Ras-expressing cells deficient in Atg5 or Atg7 exhibit an accumulation of internalized macropinocytic cargo, albumin, or dextran in macropinosomes, rather than the complete degradation by lysosomal enzymes, indicating that autophagy may degrade proteins taken up from the extracellular environment during macropinocytosis, particularly in an Atg5- or Atg7-dependent manner.

When mTORC1 activity is inhibited pharmacologically or genetically by treatment with rapamycin or knock-down of raptor, an essential component of the mTORC1 complex, macropinocytosis becomes more active, showing increased uptake of dextran and BSA in addition to activated autophagy. Moreover, cellular growth defects due to nutrient starvation are reversed more efficiently by treatment with albumin when mTORC1 is inhibited.

Based on this reciprocal regulation of mTORC1 by catabolic pathways and depending on the tumor environment, mTORC1 inhibition could enhance the efficacy of anti-tumorigenic therapies in combination with targeting macropinocytosis or autophagy. Accordingly, in vivo xenograft experiments using a PDAC cell line demonstrate that the concomitant manipulation of macropinocytosis/autophagy and mTORC1 confers a synergistic anti-tumorigenic effect.

In summary, mTORC1 plays a pivotal role in controlling metabolic balance, depending on tumor micro-environmental nutrient status. Understanding mutual regulatory mechanisms between mTORC1 and the catabolism of extracellular proteins could provide novel insights into the development of combinatorial therapeutics against oncogenic Ras-mediated cancers.
